# Wide Acoustic Bandgap Solid Disk-Shaped Phononic Crystal Anchoring Boundaries for Enhancing Quality Factor in AlN-on-Si MEMS Resonators

**DOI:** 10.3390/mi9080413

**Published:** 2018-08-18

**Authors:** Muhammad Wajih Ullah Siddiqi, Joshua E.-Y. Lee

**Affiliations:** 1 Department of Electronic Engineering, City University of Hong Kong, Kowloon, Hong Kong, China; josh.lee@cityu.edu.hk; 2 State Key Laboratory of Millimeter Waves, City University of Hong Kong, Kowloon, Hong Kong, China

**Keywords:** microelectromechanical systems (MEMS), AlN-on-Si resonators, phononic crystal, anchor loss, quality factor, acoustic bandgap

## Abstract

This paper demonstrates the four fold enhancement in quality factor (Q) of a very high frequency (VHF) band piezoelectric Aluminum Nitride (AlN) on Silicon (Si) Lamb mode resonator by applying a unique wide acoustic bandgap (ABG) phononic crystal (PnC) at the anchoring boundaries of the resonator. The PnC unit cell topology, based on a solid disk, is characterized by a wide ABG of 120 MHz around a center frequency of 144.7 MHz from the experiments. The resulting wide ABG described in this work allows for greater enhancement in Q compared to previously reported PnC cell topologies characterized by narrower ABGs. The effect of geometrical variations to the proposed PnC cells on their corresponding ABGs are described through simulations and validated by transmission measurements of fabricated delay lines that incorporate these solid disk PnCs. Experiments demonstrate that widening the ABG associated with the PnC described herein provides for higher Q.

## 1. Introduction

Quartz crystal resonators are integral components in existing radio frequency (RF) communication systems. With the advantages of small form factor and CMOS process compatibility, microelectromechanical systems (MEMS) resonators have been of interest for providing integrated clock-chip solutions. Among MEMS resonators, piezoelectric-on-silicon resonators have the advantage of possessing more efficient electromechanical coupling (in relation to capacitive silicon resonators), lower acoustic loss and high power handling capacity (in relation to piezoelectric film body resonators) [1]. High Q and efficient coupling lead to lower motional resistance (*R_m_*). As high Q and low *R_m_* are desired for low phase noise oscillators [2], various approaches have been proposed to improve the Qs (thus also reducing *R_m_*) of different piezoelectric resonators by suppressing anchor losses. This includes using biconvex plates to trap acoustic energy in the center of the acoustic cavity and thus minimizing the distribution of energy closed to the anchors [3]. For AlN-body resonators, butterfly-shaped AlN plates have been proposed [4]. Another approach has been to etch acoustic reflectors into the anchoring boundaries of the resonator to reflect the outgoing acoustic waves from the supports back into the resonator [5]. Moreover, phononic crystals (PnCs) have been hybridized with Silicon (Si) [6], Aluminum Nitride (AlN) [7,8], AlN-on-Si [9,10,11,12] and Gallium Nitride (GaN) [13] resonators with either one-dimensional (1D) or two-dimensional (2D) periodicity. PnCs are inhomogeneous periodic structures (e.g., solid–air) that shape the transmission of phonons through the PnC. This ability to shape the behavior of acoustic waves in the PnC is due to the generation of an ABG that arises from the periodic alternation of acoustic properties (velocity and density) in the PnC. From the literature, PnCs of different shapes and geometries have been investigated in relation to their effect on the width of the associated ABG. The more common PnC shapes proposed in the literature include air-holes in substrate [11,14,15,16,17,18,19], rings [9,10], cross-shaped [7,20], snowflake air inclusions [21] and fractals [22]. When a defect is introduced in a periodic PnC structure, various resonators and waveguides may be formed. PnCs can be engineered with a desired bandgap around a target center frequency and can be incorporated either in 1D or 2D patterns to realize cavity resonators and waveguides. The PnCs around the cavity behave as an acoustic shield that confines the acoustic energy within the cavity. However, cavity resonators and waveguides are associated with lower Qs and higher insertion losses. Alternatively, PnC based supporting tethers [6,7,8,9,10,11] have been used in lieu of simple beam tethers in resonators to reduce anchor loss and thus enhancing Q. As propagation of acoustic waves at frequencies within the ABG is prohibited within the PnC [23], placing PnCs close to the resonator where acoustic waves are expected propagate out has the benefit of reducing anchor loss. On this note, the size of the ABG depends on the shape of the PnC unit cell. 

Using simulations based on AlN as the solid matrix, Ardito [24] showed that shaping the PnC unit cell as a solid-disk produces a notably wider ABG compared to other more common shapes. This interesting PnC design resulted from an optimization process through applying the Bidirectional Evolutionary Structural Optimization (BESO) technique. Recently, we applied the solid-disk PnC unit cell topology proposed by Ardito [24] to a piezoelectric AlN-on-Si Lamb mode resonator to demonstrate the proof of concept [25].

Most reports of the use of PnCs to enhance Q of piezoelectric resonators have been on AlN-body resonators where the enhanced Qs have been rather limited [7,8]. While there has been some coverage of PnCs applied to AlN-on-Si resonators, the enhanced Qs have been limited to below 4000 [9,10,11,12]. In comparison, we have previously demonstrated Qs around 10,000 using biconvex resonators to reduce anchor loss to the point where electrode-related losses become dominant [26] but not with PnCs to date [27]. This work seeks to validate the effectiveness of the uniquely optimized PnC design proposed by Ardito [24] in reaching similar levels of Q. We show that these PnCs are able to enhance Qs to the same levels as the biconvex resonators reported in [26]. These PnC enhanced resonators also show similar Qs with respect to the number of electrodes, thus verifying that the PnCs can likewise reduce anchor to a point where other kinds of losses related to the electrodes become dominant. However, compared to the biconvex resonators, the use of PnCs anchors avoids the need to modify the resonator shape and compromise on the coupling area. 

This work extends from the preliminary work reported in [25] by experimentally validating the existence of a wide band gap. By incorporating the PnC into a delay line, we demonstrate a stop band that 120 MHz wide around a center frequency of 141 MHz. Furthermore, we demonstrate how adjustments to the dimensions of the PnC cell affect the size of the band gap. These wide ABG PnCs were applied as the anchoring boundaries of 7th-order mode AlN-on-Si Lamb mode resonators with resonant frequencies of around 144 MHz to demonstrate enhancement in Q of over 4-fold.

## 2. Design and Simulations of ABG 

**Figure 1:**
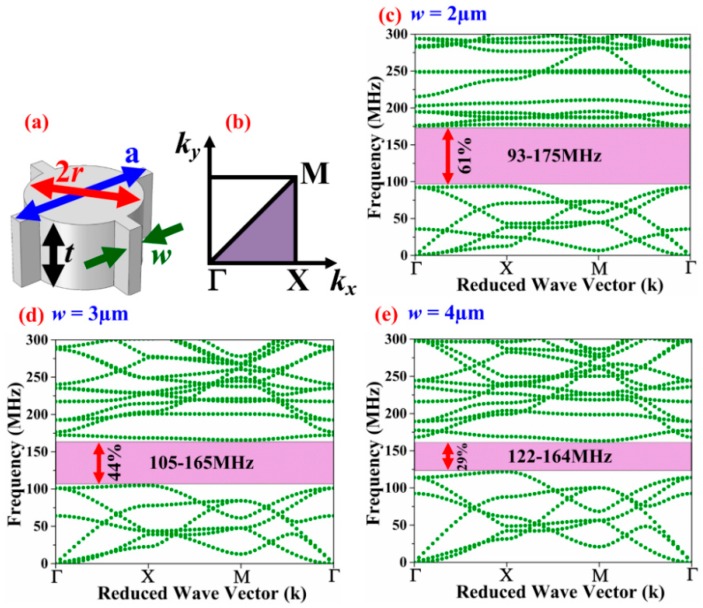
(**a**) Perspective view of solid disk PnC basic unit cell; (**b**) 1st irreducible Brillouin zone in k-space, frequency band diagrams by finite element (FE) analysis of a solid disk PnC for various inter-cell link widths of (**c**) *w* = 2 μm (**d**) *w* = 3 μm (**e**) *w* = 4 μm. Figures modified from the preliminary work in [25].

As depicted by Figure 1a, the basic PnC unit cell has a lattice constant, *a* = 22 μm and comprises a solid disk with a radius, *r* = 8 μm, with a thickness, *t* = 10 μm (set by the fabrication process [28]). The solid disk in a given unit cell is linked to the solid disks in adjacent cells by slender links. The minimum width of these links, *w* = 2 μm, is limited by the fabrication process. Using COMSOL Multiphysics (version 5.2a, COMSOL Co., Ltd., Shanghai, China) finite element (FE) analysis, the phononic band structure was computed by applying Floquet periodic boundary conditions on the unit cell along the x- and y-axis and performing a parametric sweep in the wave vector *k* bounding the first Brillouin zone illustrated by Figure 1b. The lattice parameter and disk radius were tuned in the FE simulations to synthesize a wide complete ABG for which the center frequency of the band coincides closely with the resonant frequency of the Lamb mode resonator to be physically bound by the PnC. The size of the ABG can be expressed in terms of a dimensionless parameter using the gap-to-mid gap ratio to avoid frequency dependence:

(1) $$\mathrm{BG}=\frac{f_{U}-f_{L}}{(f_{U}+f_{L})/2}$$

where *f_U_* and *f_L_* are the upper and lower bounds, respectively, of the ABG. The widest complete ABG of 82 MHz spanning from 93–175 MHz (depicted in Figure 1c) was obtained when the inter-cell link was set to its minimum (i.e., *w* = 2 μm), which corresponds to a bandgap ratio of 61%. Increasing *w* while keeping all other geometrical parameters unchanged, the ABG was reduced to 44% when *w* = 3 μm, and further down to 29% when *w* = 4 μm. As such, the simulations show that widening the inter-cell link reduces the ABG. Table 1 compares the simulated ABG of the proposed solid disk PnC with the commonly used air-hole, ring, cross inclusion, square hole and fractal PnCs assuming the same lattice size *a* = 22 μm and minimum feature size of 2 μm. The dimensions of each PnC shape have been chosen to yield close to the widest possible ABG. The center frequencies of the associated ABGs are within a similar range. A more exact alignment of center frequencies would require adjustments to the lattice parameter and various dimensions in each PnC cell, although the ultimate conclusion on which shape provides the widest ABG remains the same. The proposed solid-disk PnC possesses the highest gap-to-mid gap ratio (BG).

**Figure 2:**
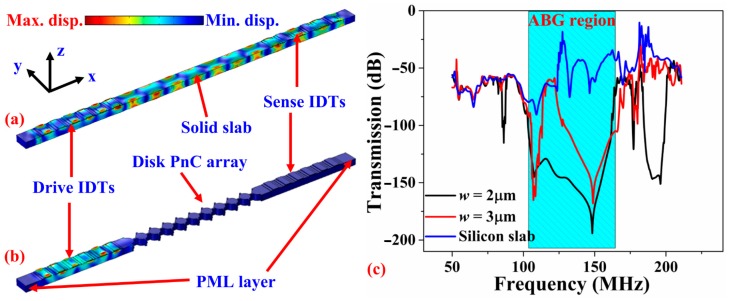
FE simulations with periodic boundary conditions applied along the y-axis—Displacement profiles of the (**a**) reference delay line having solid slab as the transmission medium and (**b**) delay line with PnCs (with inter-cell link width *w* = 3 μm) as the transmission medium. (**c**) Simulated transmission S_21_ of the three delay lines showing the existence of the bandgap associated with the solid disk PnC observed through significant drops in transmission within a wide stop band.

Next, FE models to simulate the effect of the PnC (and absence of) on the transmission through a delay line were considered to compare against the experimental results. Figure 2a,b shows the 3D FE simulated displacement profiles of the control delay line having solid slab as transmission medium and delay line with PnCs as transmission medium between drive and sense interdigitated transducers (IDTs) respectively. To reduce computational time, the frequency response simulations were carried out on a single row of 10 solid-disk PnCs with 6 finger electrodes for the IDTs on either side. Periodic boundary conditions were applied in the transverse direction (i.e., y-axis with reference to coordinate system annotated in Figure 2a,b. In the control delay line the PnCs were replaced by a solid slab. To minimize the effect of the reflected waves, Perfectly Matched Layers (PMLs) were introduced at the ends of the delay lines in the longitudinal direction. The FE simulation result illustrates the drop in transmission at a frequency range that corresponds to the associated ABG of the PnC as the PnC transmission medium effectively blocks the propagation of waves generated from the drive IDTs for frequencies that lie within the ABG that otherwise propagate through.

## 3. Experimental Validation of ABG

**Figure 3:**
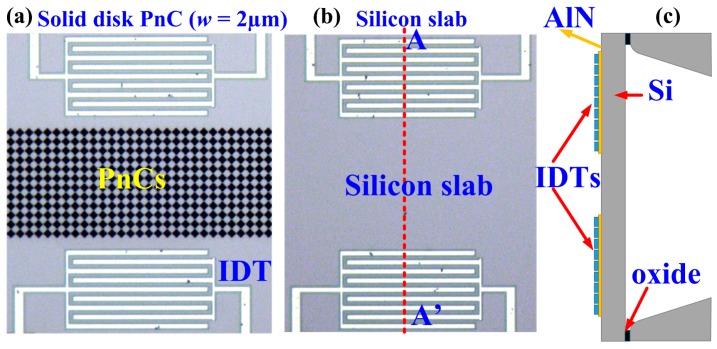
Micrographs of the fabricated delay lines incorporating (**a**) PnCs (with link width of 2 μm) for the transmission medium and (**b**) solid silicon slab as the transmission medium (as the control device for comparison). (**c**) Transverse-view schematic of the delay line showing that the regions with IDT electrodes are released for both types of delay lines.

To experimentally verify the existence of the simulated ABGs associated with the solid-disk PnCs, we designed and fabricated three delay lines using a standard AlN-on-SOI MEMS process [28], two of which are depicted in Figure 3. The delay lines comprise a pair of IDTs with a pitch of 22 μm on each side of the delay line and an aperture of 22 μm. In two of the delay lines, 12 rows of solid disk PnCs were etched between the IDTs. Figure 3a shows one of these delay lines with solid disk PnCs in the transmission medium. The PnCs either had inter-cell link widths of *w* = 2 μm or *w* = 3 μm. The third delay line, depicted in Figure 3b, serves as a reference device by incorporating just a solid silicon slab as the transmission medium between the IDTs. As shown in the side view schematic in Figure 3c, the regions with IDT electrodes are released for both types of delay lines. 

**Figure 4:**
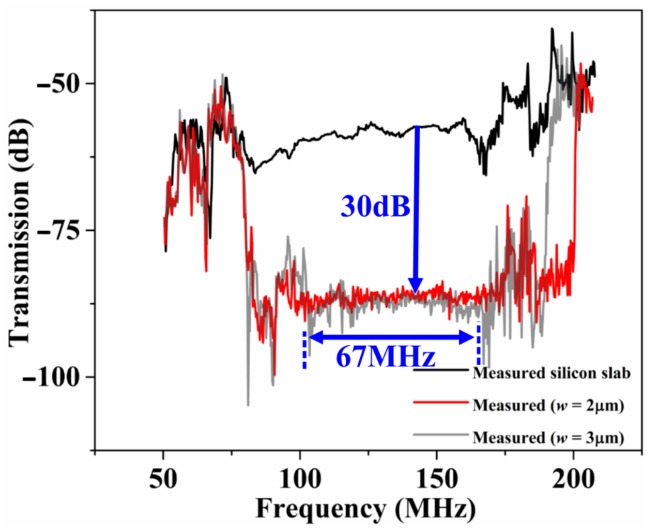
Measured transmission S_21_ of the three delay lines showing the existence of the bandgap associated with the solid disk PnC observed through the 30 dB drop in transmission within a wide stop band.

In the experiments, to reduce parasitic feedthrough, a fully-differential probe configuration was applied to the IDTs. The measured transmission (S_21_) curves from the delay lines with two different PnCs and a solid silicon slab as the propagating medium are shown in Figure 4. From Figure 4, we see that the delay lines incorporating the solid disk PnC both show an abrupt drop in S_21_ in the form of a wide band-stop filter. The drop in S_21_ measured around the middle of the stop band was 30 dB. The experimental transmission response of the PnC delay lines generally agree with the FE simulated response. The difference in the amount of attenuation between experiments and simulation within the stop band may be due to the difference between employing a finite number of rows (in the actual device) as opposed to an infinite lattice (assumed in the FE simulations in relation to the applied periodic boundary conditions). From the experiments, we see that the PnC with the narrower inter-cell links (*w* = 2 μm) yielded a wider stop band compared to the wider inter-cell link (*w* = 3 μm). 

## 4. Experimental Validation of Q Enhancement with PnC Anchors

### 4.1. Resonators with Partial Coverage of Three IDT Fingers.

**Figure 5:**
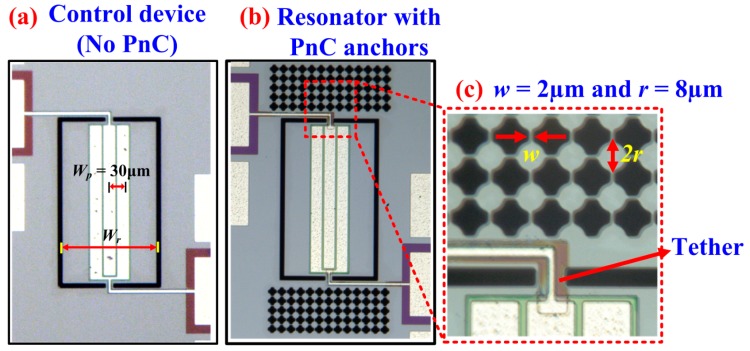
Optical micrographs of the fabricated devices partially covered with 3 IDT fingers: (**a**) Control device with no PnCs, (**b**) PnC bounded resonator, and (**c**) close-up view of the PnC matrix in relation to the Lamb mode resonator connected through the tether whose width was kept to the minimum allowed by the process.

Having verified the existence of the wide ABG associated with the solid disk PnC, we applied the PnCs as anchoring boundaries to a rectangular plate resonator to demonstrate their effectiveness in enhancing Q by reducing anchor loss. The resonator has a center-to-center electrode pitch *W_p_* = 30 μm (as shown in Figure 5a), designed to be transduced in the 7th-order symmetric Lamb mode that occurs at a frequency of 141 MHz, though lower harmonic modes can be transduced as well (as will be seen in Section 4.3). The resonant frequency for any given harmonic mode is described by:

(2) $$f=\frac{n\upsilon }{2W_{r}}$$

where *v* is the acoustic velocity of the resonator, *W_r_* is the width of the resonator, and *n* is the mode number of the respective harmonic. Hence to preferentially transduce the 7th-order Lamb mode, *W_p_* = *W_r_*/7. As shown by the micrograph depicted in Figure 5b, the Lamb mode resonator is bound on each anchoring side by solid disk PnCs. As the frequency of the 7th-order symmetric Lamb mode lies well within the ABG of the PnCs, outgoing acoustic waves from the supporting tether are prohibited from propagating through the PnCs. Solid disk PnCs with two different inter-cell link widths were fabricated (*w* = 2 μm, *w* = 3 μm) to investigate the effect of the size of the ABG on Q. Figure 5c provides a zoom-in image of the solid disk PnCs for one of the devices in relation to the Lamb mode resonator. The device without PnCs depicted in Figure 5a served as a control device. As these devices were released by trench etching through the bulk substrate, to ensure that the boundary conditions between the control device and the resonator devices incorporating PnCs were similar, the same trench size was used for all devices. The resonators were partially covered with IDTs, specifically only three IDT fingers to reduce the effect of electrode-related losses. 

**Figure 6:**
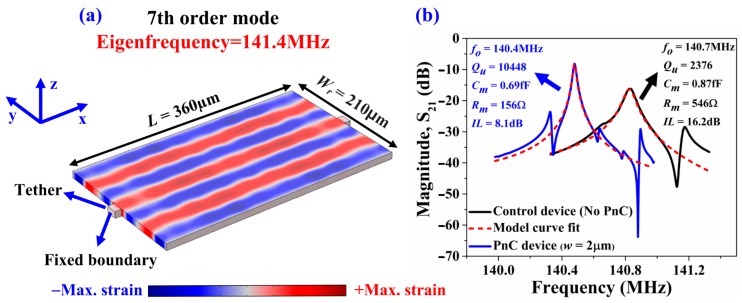
(**a**) FE simulation of the 7th-order symmetric Lamb mode where the contours denote the associated y-direction strain component. (**b**) Typical measurements of the transmission S_21_ of two of the partially covered resonators with three IDT fingers with PnC anchors (*w* = 2 μm) and a control device (no PnC anchors) for comparison to illustrate the increase in Q; the resonators were transduced in the 7th-order symmetric Lamb mode.

**Figure 7:**
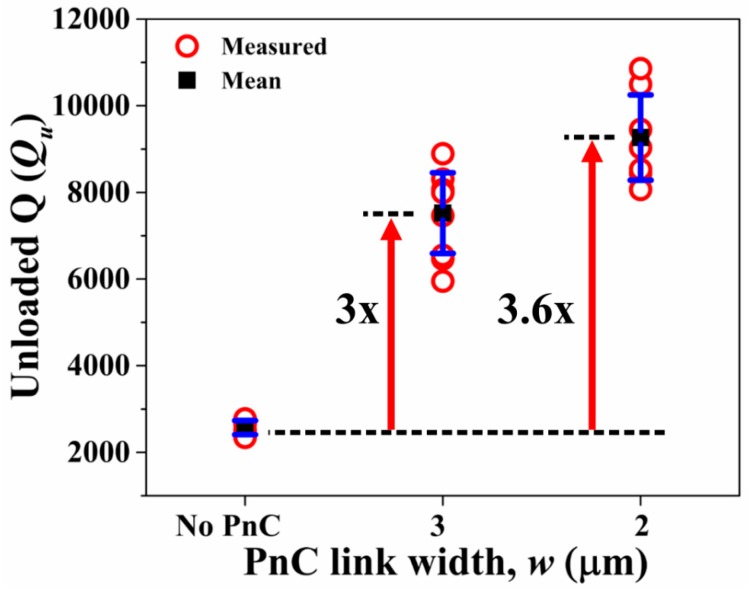
Partially covered resonators with three IDT fingers transduced in the 7th-order symmetric Lamb mode—Extracted values of unloaded Q (*Q_u_*) of the control design with no PnCs in comparison to the two other designs with PnCs in the anchors with different inter-cell link widths. The black squares denote the mean value while the error bars denote the standard deviation over six samples for each resonator design.

To experimentally validate the effect of the ABG size on the performance of the 7th-order symmetric Lamb mode AlN-on-Si resonators, we fabricated all these devices using the same AlN-on-SOI MEMS process used to fabricate the delay lines described in previous sections. The width of the supporting tether was kept to its practical minimum (16 μm) as allowed by the process with the aim to minimize losses through the tethers particularly when these are wide. As depicted in Figure 5b, five rows of PnCs were employed in the direction of outgoing wave from the tethers. On this note, it has been previously shown that the effectiveness of the PnCs in enhancing Q plateaus after three PnC cells [9]. Short-open-load-through (SOLT) calibration was performed prior to measure all resonators. Six samples of each of the three resonator types were tested (i.e., a total of 18 samples tested) to ensure repeatability. The measured resonant frequencies of the Lamb mode resonators coincide with the FE simulated values. Figure 6a depicts 7th-order harmonic Lamb mode simulated by FE. Fixed boundary conditions were applied to the tether faces in the yz-plane. Figure 6b shows the corresponding measured S_21_ of a resonator with PnC anchors (*w* = 2 μm) and a control device (no PnC anchors) to illustrate the increase in Q and corresponding reduction in insertion loss by incorporating the PnCs into the anchors. These results are typical of the measurements carried out over multiple samples tested for repeatability. Figure 7 shows the extracted values of unloaded Q (*Q_u_*) from the S_21_ responses of the 18 samples tested. It is worth pointing out that the values of Q measured for the control resonators are typical of the Lamb wave mode resonators we have fabricated with the same process and tested previously. We have also previously shown that the length of the supporting tether does not significantly alter the value of Q of these resonators based on experiments on multiple samples [29]. As such, the control resonators described herein do not represent particularly poorly designed resonators to yield a sub-optimal Q. As seen from Figure 7, incorporating PnCs into the anchors increases the mean *Q_u_* relative to the control device as much as 3.6-fold. The extent of enhancement in *Q_u_* increases with narrower inter-cell links, a trend that was also observed previously in the case air-hole in substrate PnCs [11]. For reference, the levels of Q attained using PnCs with the narrower links are similar to the 7th-order biconvex resonators of the same frequencies with three IDT fingers described in [26].

### 4.2. Resonators with Full Coverage of Seven IDT Fingers.

**Figure 8:**
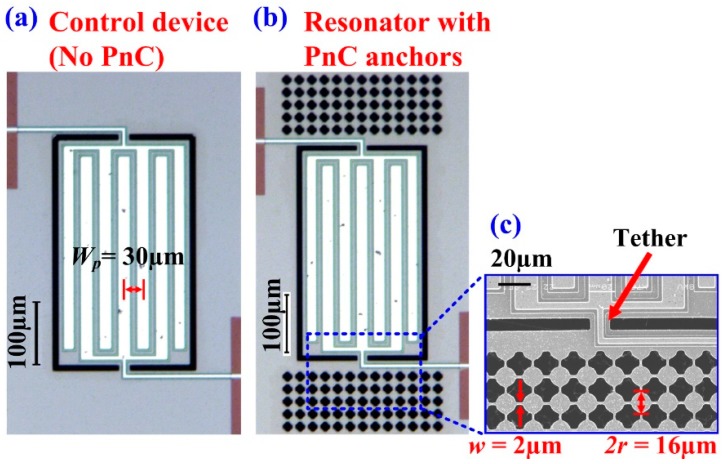
Optical micrographs of the fabricated devices: (**a**) Control device with no PnCs, (**b**) PnC bounded resonator, and (**c**) close-up view of the PnC matrix in relation to the Lamb mode resonator connected through the tether whose width was kept to the minimum allowed by the process.

To investigate the effect of the electrodes on the degree of Q enhancement when applying the PnCs in the anchoring boundaries, we designed and fabricated another set of resonators with same size of those in the previous section but with full coverage of IDT fingers (i.e., seven IDT fingers). As in the previous set of resonators with partial coverage of IDT electrodes, we considered four designs: a control device with no PnCs, and three devices with PnCs comprising different inter-cell link widths (*w* = 2 μm, *w* = 3 μm, *w* = 4 μm) to investigate the effect of the size of the ABG on Q in the limit where electrode-related losses are more dominant. Figure 8a depicts a micrograph of the control device with full IDT electrode coverage, and Figure 8b depicts a micrograph of one of the devices with PnCs anchors. Figure 8c provides a zoom-in image of the solid disk PnCs for one of the devices in relation to the Lamb mode resonator with full IDT electrode coverage.

**Figure 9:**
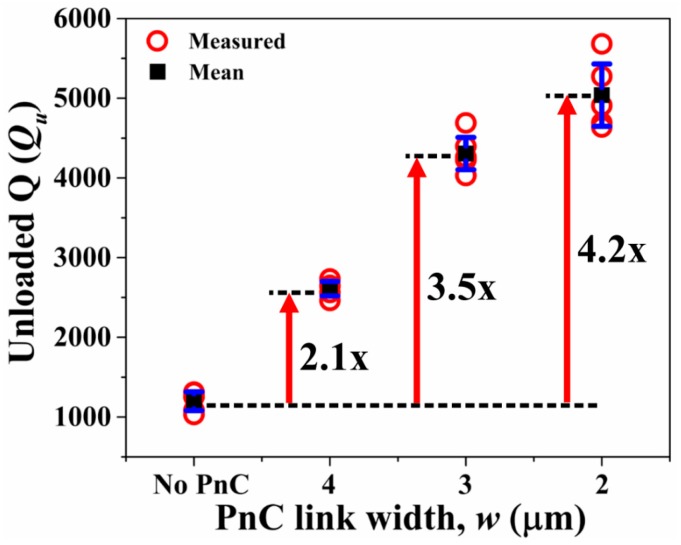
Fully covered resonators with seven IDT fingers transduced in the 7th-order symmetric Lamb mode—Extracted values of unloaded Q (*Q_u_*) of the control design with no PnCs in comparison to the three other devices with PnCs in the anchors with different inter-cell link widths. The black squares denote the mean value while the error bars denote the standard deviation over five samples.

To experimentally validate the effect of the ABG size on the performance of the 7th-order mode AlN-on-Si resonators with seven IDT fingers, five samples of each of the four resonator types were tested (i.e., total of 20 samples tested) to ensure repeatability. The measured resonant frequencies of the Lamb mode resonators are in agreement with the FE simulated values. Figure 9 depicts the extracted values of unloaded Q (*Q_u_*) from the S_21_ responses of the 20 samples tested. As seen from Figure 9, incorporating PnCs into the anchors increases the mean *Q_u_* relative to the control device by as much as 4.2-fold. As with the case of the resonators partially covered with IDT electrodes, we also see that reducing the PnC link width results in an increase in Q. But we can also see that increasing the number of IDT fingers has reduced the maximum achievable level of Q. For reference, the levels of Q attained using PnCs with the narrowest links are similar to the 7th-order biconvex resonators of the same frequencies with 5 IDT fingers described in [26]. As such, the levels of Q and trends of the resonators bounded by the widest ABG PnCs (i.e., the narrowest links) are what have been observed previously in biconvex resonators. As such, the results reported herein demonstrate that the wide ABG solid-disk PnCs are able to reduce anchor loss to a point whereby electrode-related losses begin to dominate over anchor loss.

### 4.3. Frequency Selectivity of Q Enhancement.

**Figure 10:**
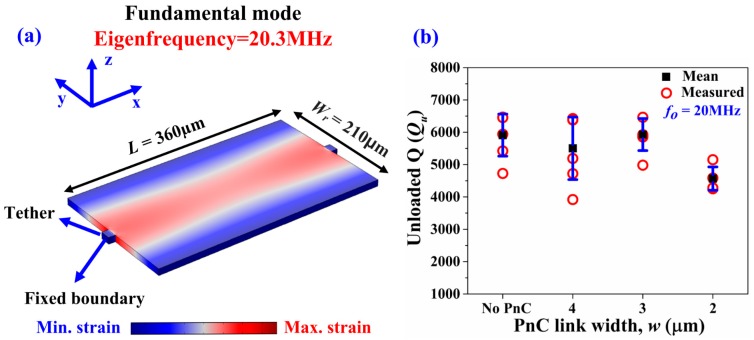
(**a**) FE simulation of the fundamental Lamb mode (occurring around 20.3 MHz) where the contours denote the associated y-direction strain component. (**b**) Fully covered resonators with seven IDT fingers—Extracted values of unloaded Q (*Q_u_*) of the control design with no PnCs compared to the other three designs with PnCs in the anchors with different inter-cell link widths transduced at the fundamental Lamb mode (20.3 MHz), which lies outside the ABG. The black squares denote the mean value, while the error bars denote the standard deviation over four samples.

**Figure 11:**
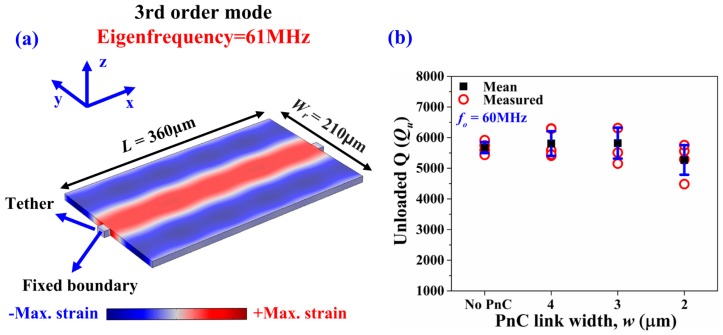
(**a**) FE simulation of the 3rd-order symmetric Lamb mode (occurring around 61 MHz), where the contours denote the associated y-direction strain component. (**b**) Fully covered resonators with seven IDT fingers—Extracted values of unloaded Q (*Q_u_*) of the control design with no PnCs compared to the other three designs with PnCs in the anchors with different inter-cell link widths transduced at the 3rd-order symmetric Lamb mode around 61 MHz that lies outside the ABG. The black squares denote the mean value while the error bars denote the standard deviation over four samples.

To show that the effect of Q enhancement applies to frequencies that lie within the ABG, we tested the very same resonators at their fundamental mode (20.3 MHz) and 3rd-order mode (61 MHz). Both these frequencies lie outside the theoretical bandgap of the same PnC topology but with different inter-cell link widths. We tested four die samples for each of the four designs (i.e., 16 devices in total). Figure 10a depicts the fundamental Lamb mode simulated by FE (occurring around 20.3 MHz), while Figure 10b summarizes the associated unloaded Qs extracted from the measurements. Similarly, Figure 11a depicts the 3rd-order Lamb mode simulated by FE (occurring around 61 MHz), while Figure 11b summarizes the associated unloaded Qs extracted from the measurements of the very same devices. We see that, for either harmonic mode, the PnCs do not provide any enhancement of Q. 

Table 2 compares the performance of the PnC resonator hybrids (3 IDTs and inter-cell link widths of 2 μm) transduced in the 7th-order symmetric Lamb mode reported herein with other state-of-the-art piezoelectric AlN and AlN-on-Si resonators disclosed in the literature for similar resonant frequencies.

## 5. Conclusions

In conclusion, we have experimentally demonstrated the existence of a wide ABG associated with a solid-disk PnC unit cell topology. By incorporating these PnCs into a delay line, we have experimentally demonstrated a bandgap ratio of 85%, inferred from the transmission measurement of a delay line incorporating such PnCs. Moreover, we have shown that the geometrical dependence of the bandgap ratio predicted by FE simulations is corroborated by the experiments. We have applied these solid-disk wide ABG PnCs to the anchors of AlN-on-Si Lamb mode resonators to demonstrate enhancements in Q by fourfold. The geometric dependence of the ABG is also evident in the extent of enhancement in Q provided by the PnC. 

## Figures and Tables

**Table 1:** Comparison of simulated ABGs between various PnC shapes using the same lattice parameter *a* = 22 μm and minimum feature size of 2 μm.

PnC Shape	Dimensions (μm)	ABG Range (MHz)	*f_c_* (MHz)	BG (%)
Air-Hole	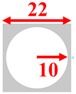	136–147	141.5	7.7
Ring	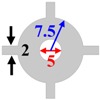	107–144	125.5	29.4
Cross Inclusion	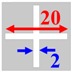	89–147	118	49.1
Square Hole	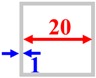	-	-	-
Fractal	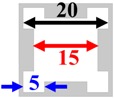	146–192	169	27.2
Solid-Disk (this work)	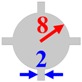	93–175	134	61.1

**Table 2:** Performance comparison of proposed PnC/resonator hybrids with the state-of-the-art piezoelectric AlN and AlN-on-Si resonators that share similar resonant frequencies.

Reference	Technology	Frequency (MHz)	Q	*f∙*Q (10^11^ Hz)
[30]	AlN	175	1500	2.6
[31]	AlN	101.71	1257	1.27
[2]	AlN-on-Si	106	4000	4.24
[27]	AlN-on-Si	100	5369	5.36
[10]	AlN-on-Si	178	1400	2.49
[9]	AlN-on-Si	141.5	5730	8.10
This work (w/o PnC)	AlN-on-Si	140.9	2510	3.53
This work (with PnC)	AlN-on-Si	140.5	10,492	14.7
